# The relationship between vitamin C or thiamine levels and outcomes for severe sepsis patients admitted to the ICU

**DOI:** 10.1038/s41598-021-94473-1

**Published:** 2021-07-23

**Authors:** Nandan Prasad, Anne V. Grossestreuer, Nuala J. Meyer, Sarah M. Perman, Mark E. Mikkelsen, Judd Hollander, David F. Gaieski

**Affiliations:** 1 Department of Emergency Medicine, Sidney Kimmel Medical College at Thomas Jefferson University, 1025 Walnut Street, Suite 300, Philadelphia, PA 19017 USA; 2 Center for Resuscitation Science, Beth Israel Deaconess Medical Center, Boston, MA USA; 3 Division of Pulmonary Critical Care Medicine, Perelman School of Medicine at the University of Pennsylvania, Philadelphia, PA USA; 4 Department of Emergency Medicine, University of Colorado School of Medicine, Aurora, CO USA

**Keywords:** Infectious diseases, Risk factors

## Abstract

Preliminary data have produced conflicting results regarding whether initial vitamin C levels in patients with severe sepsis correlate with mortality outcomes. We hypothesized that low plasma ascorbic acid or thiamine levels in severe sepsis patients admitted from the Emergency Department (ED) to the Intensive Care Unit (ICU) would be associated with increased mortality and an increased incidence of shock. Retrospective analysis of a prospective database of severe sepsis patients admitted to the ICU at an urban, academic medical center. Ascorbic acid and thiamine levels were analyzed in relation to survivors vs. non-survivors and shock vs. non-shock patients. 235 patients were included; mean age, 59.4 years ± 16.8 years; male, 128 (54.5%); in-hospital mortality, 16.6% (39/235); mean APACHE3 score, 61.8 ± 22.8; mean ascorbic acid level (reference range 0.40–2.10 mg/dL), 0.23 mg/dL (95% CI 0.07–4.02); and the mean thiamine level (reference range 14.6–29.5 nmol/L), 6.0 nmol/L (95% CI 4.0–9.5). When survivors were compared to non-survivors, survivors were more likely to be male (57.7% [113/196] vs. 38.5% [15/39]) and have lower APACHE3 scores (58.2 ± 22.6 vs. 79.9 ± 16.0). For the total cohort of 235 patients, there was no statistically significant relationship between a patient’s initial ascorbic acid or thiamine level and either survival or development of shock. In this analysis of early plasma samples from patients with severe sepsis admitted from the ED to the ICU, we found that mean ascorbic acid and thiamine levels were lower than normal range but that there was no relationship between these levels and outcomes, including 28 day mortality and development of shock.

## Introduction

Sepsis, the syndrome of life-threatening dysregulated immune response to infectious pathogens, is both common and deadly^1,2^. Early screening and identification, aggressive resuscitation, and administration of appropriate antibiotics are associated with lower mortality in patients with severe sepsis^3–10^. However, the search for effective adjuvant therapies continues. Potential candidates include ascorbic acid (vitamin C) and thiamine (vitamin B1).

Vitamin C’s redox state, ascorbate, is its most common form in human cells and has important immunologic, anti-inflammatory, and physiologic properties^11^, including endogenous biosynthesis of catecholamines^12,13^, enhancement of catecholamine receptors^14,15^, and endogenous synthesis of vasopressin, a regulator of vascular tone^16^. Regarding immunologic function, Vitamin C increases lymphocyte transformation, polymorphonucleocyte motility, and serum levels of IgM and C3^17–19^. One of ascorbate’s best-known functions is as an anti-oxidant, neutralizing free radical species, which can lead to cell damage and death^20^, and helping recycle and preserve protein and lipid radicals^21^. Finally, and of particular importance in sepsis, ascorbate has been shown to modulate endothelial permeability^22^.

In non-randomized studies, administration of vitamin C to critically ill patients has been associated with an improvement in sequential organ failure assessment (SOFA) scores, decreased vasopressor dose and duration, and lower mortality^23,24^. In the CITRUS-ALI trial, there were no differences in the primary outcomes of change in organ dysfunction or markers of inflammation in septic Intensive Care Unit (ICU) patients with acute respiratory distress syndrome (ARDS) who received either high-dose vitamin C or placebo^25^. However, there was a significant decrease in the secondary outcome of mortality (29.8 vs. 46.3%; p = 0.03).

Thiamine, another essential micronutrient, is a necessary cofactor for the transfer of pyruvate, a breakdown product of glucose metabolism, into the Kreb’s cycle during aerobic metabolism to produce adenosine triphosphate (ATP). Thiamine-deficient patients produce excess lactate, have a deficiency of ATP, and must rely on anaerobic metabolism to meet their cellular energy needs. This pathophysiologic similarity to sepsis raises the question whether thiamine has a role as an adjuvant sepsis therapy. A percentage of septic shock patients are thiamine deficient^26–28^ and thiamine administration improves their lactate clearance^28^. However, a randomized, double-blind trial of thiamine administration to septic shock patients demonstrated no difference in 24-h lactate levels, shock reversal, severity of illness, or mortality^29^.

Although these preliminary studies have produced conflicting results, they have generated enthusiasm about combining vitamin C, thiamine, and hydrocortisone, which is thought to be synergistic with these essential nutrients, as a low cost treatment for septic shock. A before and after study by Marik et al. compared outcomes for 47 patients receiving the combination of hydrocortisone, vitamin C, thiamine (HAT) to 47 consecutive historic control patients who did not receive HAT and showed a 31.9% (40.4% vs. 8.5%; p < 0.01) absolute mortality reduction in the treatment group^30^. These intriguing, hypothesis-generating results led to several randomized, control trials. In the VITAMINS trial, the first, large, multicenter trial testing the efficacy of HAT, septic shock patients admitted to the ICU were randomized to HAT vs. hydrocortisone alone in an open-label, multicenter trial. No difference in time alive or vasopressor-free days during the first 7 days of hospitalization, or 90-day mortality were found^31^. The results of two additional studies yielded similar results: the ACTS trial, a randomized, double-blind trial of HAT, demonstrated no difference between the treatment group and placebo^32^; the VICTAS trial, the largest trial to date, enrolling 501 patients randomized to HAT vs. placebo, also demonstrated no difference in vasopressor and ventilator free days or in-hospital mortality^33^.

To further understand the role of vitamin C and thiamine in severe sepsis, we measured ascorbic acid and thiamine levels in banked plasma samples drawn early in the clinical course of patients admitted from the Emergency Department (ED) to the ICU with severe sepsis present on admission. The primary hypotheses of the investigation were: (1) low plasma ascorbic acid levels are associated with increased mortality at 28 days; (2) low plasma thiamine levels are associated with increased mortality at 28 days. The secondary hypotheses were: (1) low plasma ascorbic acid levels are associated with increased incidence of shock; (2) low plasma thiamine levels are associated with increased incidence of shock; (3) the presence of liver disease would worsen outcomes in patients with either low ascorbic acid or thiamine levels; (4) an interaction will exist between thiamine levels, tertiles of ascorbic acid levels, and mortality at 28 days, with lower combined essential nutrient levels correlating with worse outcomes.

## Methods

### Study design

We performed a retrospective analysis of samples from a subset of the patients enrolled in the Molecular Epidemiology of Severe Sepsis in the ICU (MESSI) study between September 14, 2008 and August 1, 2010^34^. MESSI study patients were admitted from the ED to the medical ICU (MICU) at an urban academic tertiary referral center, and were prospectively enrolled if they had ≥ 2 systemic inflammatory response syndrome criteria, a known or strongly suspected infection, and evidence of organ dysfunction or shock, following the 2nd International Sepsis Definitions criteria^35^. Liver disease was defined as having a past medical history significant for cirrhosis. Data were also collected on potential confounders that may interfere with vitamin C and thiamine metabolism including alcohol consumption, current smoking, end-stage renal disease (ESRD), and chronic kidney disease (CKD) without ESRD. Exclusion criteria included a lack of commitment to life sustaining treatment at the time of admission, primary reason for admission unrelated to severe sepsis (e.g. cardiac arrest, pulmonary embolism), transfer from an outside hospital ICU, and previous enrollment. Based on an average mortality for severe sepsis patients at our institution of approximately 15%, a hypothesized mortality of 30% in vitamin C and/or thiamine deficient patients, a 50% vitamin deficiency rate, an alpha of 0.05, and power of 80%, we estimated that a sample size of 240 patients would be required to investigate the relationship. This study was approved by the University of Pennsylvania Institutional Review Board and granted a waiver of timely informed consent, which was obtained from patients or their surrogates as soon as possible after MICU admission. All methods were performed in accordance with relevant guidelines and regulations. In the current analysis examining the relationship between ascorbic acid levels, thiamine levels, and outcomes, we limited the MESSI cohort to patients admitted directly from the ED to the ICU.

### Data and sample collection and assay analysis

Research personnel used structured case report forms to collect data, the details of which have been reported previously^34^. Study samples were obtained in the ED at first blood draw when intravenous access was obtained, either before or at the time zero of severe sepsis diagnosis. Residual citrated plasma was collected in citrated vacutainers, immediately centrifuged, stored @ 4 °C for a maximum of 48 h before long-term storage @ − 80 °C, protected from light, until analysis of ascorbic acid and thiamine levels was performed. A mean of four years time elapsed between collection/storage and sample analysis. For each measurement, a validated reference range for healthy subjects was provided by the manufacturer. Control material concentrations were performed and low and high control ranges were assessed using previously collected samples from healthy volunteers. Analyses were performed in the Translational Core Laboratory in the Institute for Translational Medicine and Therapeutics at the Perelman School of Medicine at the University of Pennsylvania. The vitamin C levels were analyzed by High Performance Liquid Chromatography (HPLC) and performed on a Shimadzu HPLC platform. The reference range for healthy asymptomatic adults for ascorbic acid was 0.4–2.1 mg/dL (4–21 μg/mL or 22.8–119.2 μmol/L). The vitamin B1 levels were also analyzed by HPLC and performed on a Shimadzu HPLC platform. The reference range for healthy asymptomatic adults for thiamine plasma samples was 14.6–29.5 nmol/L (4.2–8.5 ng/mL).

### Data analysis

Descriptive data are presented as means with standard deviations for continuous data and frequencies and percentages for categorical data. Percent differences are presented with 95% confidence intervals (CIs). Ascorbic acid and thiamine levels were analyzed in relation to survivors vs. non-survivors, shock vs. non-shock, and in relation to whether patients had liver disease given the relationship between glycolysis, the Krebs cycle, the Cori cycle, and aerobic metabolism. We also analyzed the interaction between thiamine levels, tertiles of ascorbic acid levels, and outcomes. All analyses were performed unadjusted and adjusted for age, sex, lactate level, and APACHE 3 score.

## Results

### Demographics and outcomes

Data from a total of 235 patients and their plasma sample results were included in the study (Table 1). The mean age of the subjects was 59.4 years ± 16.8 years; 128 (54.5%) were male; in-hospital mortality was 16.6% (39/235); 13.6% (32/235) had a past medical history of liver failure; the mean APACHE3 score was 61.8 ± 22.8; 26.4% (62/235) currently consumed alcohol on a regular basis; 53.6% currently smoked cigarettes; 10.6% (25/235) had ESRD; and an additional 22.1% (52/235) had CKD without ESRD. The mean ascorbic acid level was 0.23 mg/dL (95% CI 0.07–4.02); the mean thiamine level was 6.0 nmol/L (95% CI 4.0–9.5). When survivors were compared to non-survivors, the patients who survived were more likely to be male (57.7% [113/196] vs. 38.5% [15/39]) and had lower APACHE3 scores (58.2 ± 22.6 vs. 79.9 ± 16.0).

**Table 1:** Mortality.

	Survivors (n = 196)	Non-survivors (n = 39)	p value
Age	60.2 ± 16.9	55.6 ± 16.0	0.106
Male	113 (57.7)	15 (38.5)	0.028*
APACHE3	58.2 ± 22.6	79.9 ± 23.8	< 0.001*
Thiamine (nml/L)	5.9 (3.9, 8.7)	6.7 (4.2, 13.4)	0.127
Vitamin C (mg/dL)	2.4 (0.7, 4.1)	1.9 (0.7, 3.6)	0.479
Liver Disease	27 (13.8)	5 (12.8)	> 0.999

### Relationship between ascorbic acid levels, survival, and shock

For the total cohort of 235 patients, there was no statistically significant relationship between a patient’s initial ascorbic acid level and either survival or development of shock in unadjusted (Tables 2, 3) and adjusted [not shown in Tables] analyses.

**Table 2:** Thiamine, vitamin C, and survival.

	OR	95% CI	p value
**Thiamine (nml/L)**			
Survival to discharge	0.99	0.96–1.01	0.357
28 day survival	0.98	0.95–1.00	0.159
60 day survival	0.98	0.95–1.00	0.093
One year survival	0.98	0.95–1.00	0.144
Shock	1.00	0.97–1.04	0.790
**Vitamin C (mg/dL)**			
Survival to discharge	1.05	0.91–1.20	0.521
28 day survival	0.94	0.82–1.07	0.329
60 day survival	1.02	0.89–1.16	0.782
One year survival	0.93	0.84–1.03	0.168
Shock	1.02	0.94–1.12	0.595

**Table 3:** Shock vs. no shock groups.

	Shock (n = 36)	No shock (n = 213)	p value
Age	60.9 ± 17.4	55.1 ± 16.0	0.048*
Male	16 (44.4)	121 (56.8)	0.168
APACHE3	81.1 ± 23.6	56.9 ± 22.7	< 0.001*
Thiamine (nml/L)	6.9 (3.9, 10.0)	5.9 (3.8, 8.7)	0.259
Vitamin C (mg/dL)	2.4 (1.1, 4.3)	2.3 (0.7, 4.1)	0.525
Liver failure	4 (11.1)	28 (13.2)	0.492

### Relationship between thiamine levels, survival, and shock

For the total cohort of 235 patients, there was no statistically significant relationship between a patient’s initial thiamine level and either survival or development of shock in unadjusted (Tables 2, 3) and adjusted (not shown in tables) analyses.

### Relationship between liver disease, ascorbic acid levels, thiamine levels, and survival

In the subset of 32 patients with liver disease, there were statistically significant relationships between low thiamine levels and in-hospital mortality and 28-day mortality. The statistical significance went away at 60 days and 1 year. There was no relationship between the presence of low ascorbic acid levels, liver disease and survival at 28 days, 60 days, or 1 year. However, when the ascorbic acid levels for the 203 patients *without* liver disease were analyzed, a statistically significant relationship between initial plasma ascorbic acid levels and 28-day mortality was found (OR 0.86; 95% CI 0.744–1.00, p = 0.05) (Table 4). This relationship was no longer significant at 60 days or 1 year. Similar results were obtained in multi-variant adjusted analyses (not shown in table).

**Table 4:** Liver disease.

	OR	95% CI	p value
**Thiamine (nml/L)**			
Survival to discharge	0.73	0.53–0.99	0.045*
28 day survival	0.73	0.53–1.00	0.048*
60 day survival	0.86	0.67–1.10	0.229
One year survival	0.80	0.62–1.03	0.089
Shock	1.28	0.93–1.77	0.134
**Vitamin C (mg/dL)**			
Survival to discharge	1.57	0.88–2.79	0.125
28 day survival	1.57	0.89–2.77	0.116***
60 day survival	1.39	0.92–2.09	0.114
One year survival	1.01	0.80–1.28	0.912
Shock	1.13	0.84–1.53	0.415

***No liver disease group: 0.86 (95% CI 0.744–1.00, p = 0.05).

### Relationship between thiamine and outcomes by tertile of vitamin C

No statistically significant relationship was found between thiamine levels, tertiles of vitamin C levels, survival in-hospital, at 28 days, 60 days, or 1 year or the development of shock in unadjusted (Table 5) and adjusted (not shown in table) analyses.

**Table 5:** Relationship between thiamine and outcomes by tertile of vitamin C.

**Survival to discharge**			
Tertile 1	1.00	0.96–1.05	0.823
Tertile 2	0.95	0.88–1.02	0.168
Tertile 3	0.95	0.89–1.02	0.168
**28 day survival**			
Tertile 1	1.00	0.96–1.05	0.839
Tertile 2	0.93	0.86–1.01	0.084
Tertile 3	0.89	0.78–1.00	0.058
**60 day survival**			
Tertile 1	0.99	0.96–1.02	0.468
Tertile 2	0.96	0.89–1.04	0.269
Tertile 3	0.92	0.84–1.01	0.087
**One year survival**			
Tertile 1	0.99	0.95–1.02	0.439
Tertile 2	0.97	0.90–1.04	0.389
Tertile 3	0.93	0.84–1.03	0.163
**Shock**			
Tertile 1	1.01	0.97–1.05	0.727
Tertile 2	1.00	0.91–1.09	0.926
Tertile 3	1.02	0.94–1.10	0.649

## Discussion

In this analysis of early plasma samples from critically ill patients with severe sepsis admitted to the MICU from the ED, we found that the mean vitamin C and thiamine levels were low but that there was no relationship between these initial plasma vitamin C or thiamine levels and outcomes, including mortality and the development of shock, in the total cohort. This is similar to other studies, which have demonstrated that vitamin C levels^36–42^ and thiamine levels^28^ are low in a significant percentage of critically ill patients. It is important to note that these patient cohorts examine heterogeneous groups of patients with severe sepsis or septic shock, trauma, surgical wound infections, ARDS, or who were post-operative and, in many studies, compared their anti-oxidant levels to those of healthy volunteers. In a population similar to our study cohort, Doise et al*.* measured total plasma antioxidant capacity along with serial vitamin C levels and found that patients with severe sepsis and septic shock had significantly lower vitamin C levels compared to healthy individuals, and that their levels continued to fall after the initial diagnosis^36^. Similarly, for thiamine, Donnino et al*.* found that 35% of patients with elevated lactate on ED presentation had thiamine deficiency^29^.

The mean vitamin C and thiamine levels in our cohort were 58% and 41% of the lower limit of the reference range provided by the assay manufacturers, respectively, findings which were confirmed by quality control samples run on stored samples from healthy volunteers during our calibration phase of analysis. These results are similar to Schorah et al*.*’s finding that median vitamin C levels in critically ill patients were < 25% of those obtained from healthy volunteers^39^. Additional research on the subject has found that low vitamin C levels are associated with poorer prognosis in critical illness. For example, Borrelli et al*.* followed SICU patients during their hospital course and found that those with low vitamin C levels were at higher risk of developing multi-organ failure^43^. Our data did not reproduce these findings in the cohort as a whole, where there was no correlation between the presenting vitamin C and thiamine levels and outcomes, including the development of shock.

More recent data regarding vitamin C and, in a subset of the studies, thiamine levels, are available from 4 recent studies—Marik et al.’s before and after study^30^, and the ORANGES, VICTOR, and ATESS trials^44–46^. In Marik et al.’s study “accurately timed” initial vitamin C levels were available for 22/47 (46.8%) patients in the treatment group, the mean level was significantly below the lower limit of normal, and no patient had a normal level^30^. This would suggest that when clinical criteria for severity of illness in sepsis are met, obtaining a baseline vitamin C level is unnecessary. In the ORANGES trial, the investigators found that 50% of the participants were vitamin C deficient and 14% had severe hypovitaminosis^44^. These deficiency levels were less severe than reported by Marik et al. and the CITRUS-ALI trial investigators^25,30^ and the ORANGES investigators found no difference in in-hospital mortality or ventilator-free days when patients were treated with HAT. However, HAT therapy did significantly reduce the time to shock resolution. In the VICTOR trial^45^, the baseline values of vitamin C were similar to what we reported in our study, 1.49 μg/mL [0.15 mg/dL] and 2.1 μg/mL [0.21 mg/dL] in the routine therapy and HAT groups, respectively, vs. 0.23 mg/dL in our study population. The authors comment that their baseline levels are significantly lower than those reported in the ORANGES, Marik et al., and CITRUS-ALI trials^45^. Finally, the ATESS trial investigators found that 50.9% of the HAT group and 47.3% of the placebo group were vitamin C deficient at the time of enrolment in the trial and that the median vitamin C level in the HAT group increased significantly over the first 72 h of therapy^46^. Thiamine deficiency in baseline samples was much less common, occurring in 9.4% of the HAT group and 7.0% of the placebo group^46^. These data, taken as a whole, suggest that illness severity, not vitamin C and thiamine levels, should inform the implementation of HAT therapy, if it is used at all.

Further, when the subset of the overall MESSI cohort examined in our study was limited to patients with end-stage liver disease, there were no differences in outcomes in these patients when analyzed in relation to initial vitamin C or thiamine levels. An interesting result emerged in this analysis: the mortality of patients *without* end-stage liver disease correlated with their initial Vitamin C levels with lower levels associated with higher mortality at 28 days. These results do not provide information about whether repletion with therapeutic or supra-therapeutic doses of Vitamin C would improve outcomes for these patients. This finding also does not address the question of whether future studies of vitamin C as a therapeutic for septic shock should exclude patients with end-stage liver disease because of therapeutic ineffectiveness nor does it provide information about the efficacy of the use of HAT to treat this cohort of critically ill infected patients. Some researchers and clinicians have concluded that the aggregate results of the HAT trials reported to date suggest that Marik et al.’s findings have not been replicated and that no role exists for HAT therapy in the optimization of resuscitation and management of critically ill sepsis patients. Others have suggested that the HAT trials all have significant limitations. For example, Marik criticized the 12.1 h (IQR 5.7–19.0 h) median time from meeting eligibility criteria to first dose of vitamin C in the intervention group of the VITAMINS trial as “ethically and morally unacceptable^47^,” stating that the therapy was given too late to have any possible therapeutic benefit. However, in the Marik et al. trial^30^, timing of HAT therapy is described simply as: “treated with [HAT] within 24 h of ICU admission.” Secondary outcome results from the CITRUS-ALI study suggest that disease severity may be more important than exact timing of vitamin C administration for improvement in outcome: the 28 day mortality in the Vitamin C group vs. placebo was 29.8% vs. 46.3% (p = 0.03) and the number of ventilator-free days was 13.1 vs. 10.6 (p = 0.15). Further studies of patient selection for and timing of HAT therapy are needed.

Our study has several limitations. First, the study was performed at one institution and limited to patients admitted from a busy academic ED to a single MICU. Therefore, the results we found in our vitamin C and thiamine samples may not be generalizable to samples collected in different patient populations with different levels of disease severity. Second, some data suggest that serum samples of vitamin C are preferred over plasma samples. However, analysis of healthy specimen plasma samples on the Shimadzu HPLC platform provided accurate results, suggesting our plasma sample results are reliable. Third, we did not collect data on vitamin C or thiamine repletion in our cohort of patients; however, the samples were collected years before the Marik et al*.* results were published, prior to when vitamin C and thiamine repletion in critically ill sepsis patients became more prevalent^30^. This suggests that it is likely that only a small percentage of the subjects received repletion of either supplement.

### Interpretation

In this analysis of early plasma samples from patients with severe sepsis admitted to the MICU from the ED, we found no relationship in the cohort as a whole between initial plasma vitamin C or thiamine levels and outcomes including development of shock and mortality. When the cohort was limited to patients without pre-existing liver disease, vitamin C levels but not thiamine levels correlated with outcomes at 28 days with lower levels associated with higher mortality.
